# Screening, Synthesis, and QSAR Research on Cinnamaldehyde-Amino Acid Schiff Base Compounds as Antibacterial Agents

**DOI:** 10.3390/molecules23113027

**Published:** 2018-11-20

**Authors:** Hui Wang, Mingyue Jiang, Fangli Sun, Shujun Li, Chung-Yun Hse, Chunde Jin

**Affiliations:** 1 School of Engineering, Zhejiang A&F University, Lin’an 311300, China; wh-snowis@outlook.com (H.W.); jincd@zafu.edu.cn (C.J.); 2 Key Laboratory of Bio-Based Material Science and Technology of the Ministry of Education, Northeast Forestry University, Harbin 150040, China; jiangmingyue@nefu.edu.cn; 3 Southern Research Station, USDA Forest Service, Pineville, LA 71360, USA; chse@fs.fed.us

**Keywords:** cinnamaldehyde-amino acid schiff base, screening, synthesis, antibacterial activity, QSAR

## Abstract

Development of new drugs is one of the solutions to fight against the existing antimicrobial resistance threat. Cinnamaldehyde-amino acid Schiff base compounds, are newly discovered compounds that exhibit good antibacterial activity against gram-positive and gram-negative bacteria. Quantitative structure–activity relationship (QSAR) methodology was applied to explore the correlation between antibacterial activity and compound structures. The two best QSAR models showed R^2^ = 0.9354, F = 57.96, and s^2^ = 0.0020 against *Escherichia coli*, and R^2^ = 0.8946, F = 33.94, and s^2^ = 0.0043 against *Staphylococcus aureus*. The model analysis showed that the antibacterial activity of cinnamaldehyde compounds was significantly affected by the polarity parameter/square distance and the minimum atomic state energy for an H atom. According to the best QSAR model, the screening, synthesis, and antibacterial activity of three cinnamaldehyde-amino acid Schiff compounds were reported. The experiment value of antibacterial activity demonstrated that the new compounds possessed excellent antibacterial activity that was comparable to that of ciprofloxacin.

## 1. Introduction

Antibiotic resistance is a global problem, which is limiting the treatment of microbial infection [1]. Antibiotic resistance has developed rapidly and poses a serious threat to global public health [2]. New antibiotic resistance has continuously emerged, resulting in the ineffectiveness of many original antibiotic agents, and therefore, infection continues to endanger patients [3]. Furthermore, the discovery of a new antibiotic and the process to bring it to market requires approximately ten years. Therefore, there is an urgent need to find novel antibacterial agents to counter pathogenic microorganisms [4].

Schiff base, firstly synthesized in 1864 by Schiff, is a nitrogen analog of an aldehyde or ketone in which the carbonyl group (C=O) has been replaced by an imine or azomethine group [5]. The bioactivity of Schiff base compounds and its metal complexes are of interest to researchers. Many studies have been conducted on the antifungal [6], antibacterial [7], antitumor, and anti-inflammatory [8] properties of Schiff base compounds and its metal complexes.

Cinnamaldehyde is a natural antimicrobial agent and is the main component of cinnamon oil. It has been reported that cinnamaldehyde is known to inhibit the growth of fungi and bacteria, such as *Aspergillus niger*, *S. aureus* [9], and *E. coli* [10]. Furthermore, cinnamaldehyde is considered safe and has been approved by the united nations food and agriculture organization and world health organization(FAO/WHO) Expert Committee on Food Additives [11]. It is a conjugated aromatic aldehyde that can react with an amino group to generate stable Schiff base compounds [12]. Cinnamaldehyde Schiff base compounds, as the aromatic-ring, have been widely synthesized and used in the field of biomedicine because their synthesis requires only mild reaction conditions, whilst providing high reaction rates [13]. Wang et al. [14] synthesized cinnamaldehyde chitosan Schiff base and found that the antifungal activity of the cinnamaldehyde chitosan Schiff base was stronger than that of chitosan alone. Kudrat-E-Zahan et al. [15] synthesized six cinnamaldehyde dithiocarbazate Schiff base metal complexes, where the metal complexes showed strong antimicrobial activity.

Many kinds of amino have free amino groups that provide the possibility to generate cinnamaldehyde-amino Schiff base. In a previous paper, Reference [16], cinnamaldehyde-amino acids compounds were synthesized with several kinds of amino acid and cinnamaldehyde derivatives, and the resulting compounds showed comparable antibacterial activity to ciprofloxacin against *S. aureus* and *E. coli.* Furthermore, cinnamaldehyde-amino acid Schiff base compounds significantly decreased the drawbacks of cinnamaldehyde (e.g., high volatility, insolubility in water, and foul odor). The good properties of cinnamaldehyde-amino acid Schiff base compounds have proven that it has market potential. However, there are several cinnamaldehyde-amino acid Schiff base compounds and screening a compound for satisfactory bioactivity as a candidate is time-consuming work. However, the quantitative structure–activity relationship (QSAR) could determine the crucial structure factors for bioactivity using a large number of structure descriptors calculations and regression analysis [17]. QSAR models provide a significant tool to correlate antibacterial activity with the structural properties of compounds [18].

In this study, the relationship between chemical structure and the antibacterial activity of cinnamaldehyde-amino acid Schiff base compounds against *S. aureus* and *E. coli* was explored. The most important structural factors influencing the antibacterial activity were investigated. Based on the results of the QSAR models, many cinnamaldehyde-amino acid Schiff base compounds were screened using the QSAR model. Finally, three compounds with good antibacterial activity were selected to synthesize, characterize the structure, and conduct antibacterial activity testing.

## 2. Results and Discussion

### 2.1. The Study on QSAR Models

The descriptor reflects the molecular features. The screening of several significant molecular descriptors from many other descriptors was an essential procedure in the QSAR study. There are several regression methods available to establish the relationship between activity and the descriptors. In this research, best multilinear regression was used to establish the model with satisfactory statistical parameters (R^2^, F, s^2^). The best QSAR model should have a proper number of descriptors. According to the optimal multilinear regression, the number of descriptors was limited to meet the conditions of Equation (1), so as to avoid over-description of the model [19].

(1) N≥5D

**Figure 1:**
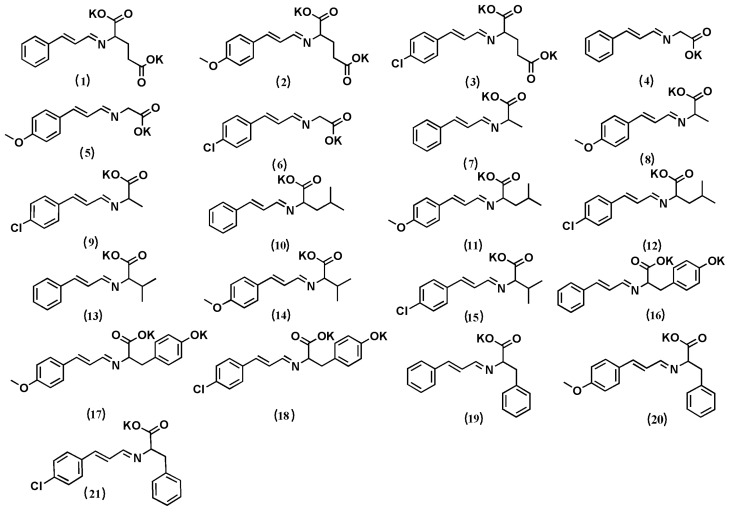
Structures of the 21 cinnamaldehyde-Schiff base compounds used to calculate the quantitative structure–activity relationship (QSAR).

N and D represent the number of samples (21 compounds) and descriptors, respectively. The sample structure used to establish the model is listed in Figure 1.

**Figure 2:**
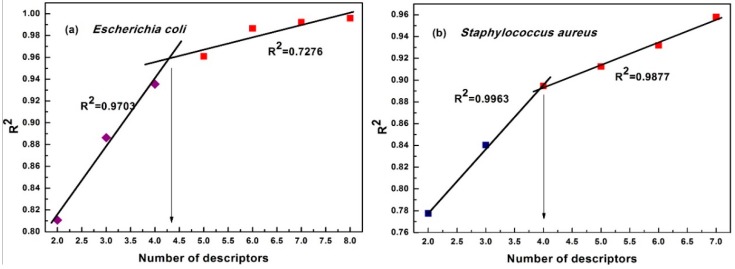
Breaking point rule for determination of the descriptor number. (**a**) is breaking point rule for model against *E. coli*, (**b**) is the breaking point rule for model against *S. aureus*.

In this research, the maximum number of descriptors was set to five. The best QSAR model should provide good statistical results and utilize the proper number of descriptors. Hence, a simple method, ‘breaking point,’ was used to determine the best QSAR model, as shown in Figure 2. In Figure 2, the increase in the number of descriptors led to a significant change in the statistical parameter, R^2^, of the regression model when the number of descriptors is less than four. Above four descriptors, the change in the R^2^ became less significant. Hence, this point was considered as the ‘breaking point’ [20], suggesting that the model that included four descriptors was the best model (The values of the descriptors are listed in Table 1). The statistics of the best QSAR model are listed in Table 2 and are also described mathematically in the following Equations (2) and (3).

(2) $$\begin{array}{cc} {\mathrm{LgAR}}_{E.c}=(5.0709 & \pm 4.0665\times 10^{-1})-\left(2.5685\pm 1.9802\times 10^{-1}\right)\times D_{1}\\ & -\left(4.1057\times 10^{1}\pm 7.4151\right)\times D_{2}-\left(1.7850\pm 3.4648\times 10^{-1}\right)\times D_{3}\\ & -\left(7.2082\pm 1.8119\right)\times 10^{-1}\times D_{4} \end{array}$$

(3) $$\begin{array}{cc} {\mathrm{LgAR}}_{S.a}=- & \left(1.8664\times 10^{1}\pm 4.3463\right)-\left(2.7525\pm 5.8814\times 10^{-1}\right)\times D_{5}\\ & \;\;\;\;\;\;\;\;\;\;+\left(2.6523\times 10^{-3}\pm 6.9487\times 10^{-4}\right)\times D_{6}\\ & \;\;\;\;\;\;\;\;\;\;+\left(1.9642\times 10^{-2}\pm 5.2856\times 10^{-3}\right)\times D_{7}\\ & \;\;\;\;\;\;\;\;\;\;+\left(1.1873\times 10^{-1}\pm 5.2091\times 10^{-2}\right)\times D_{8} \end{array}$$

**Figure 3:**
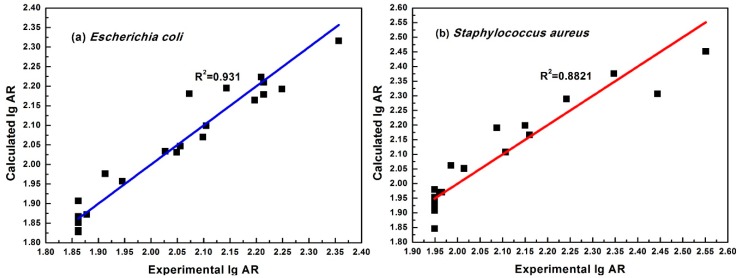
Experimental versus calculated values according to the optimal models. (**a**) is for best model against *E. coli*, (**b**) is the for model against *S. aureus*.

Equations (2) and (3) could be used to calculate the predicted antibacterial activity of 21 cinnamaldehyde compounds. The graphical relationship between the experimental lgAR (Exp.lgAR) and calculated lgAR (Cal.lgAR) is shown in Figure 3.

The validation results of the best QSAR model are presented in Table 3. The averages of the statistical results were similar for the best QSAR model. The test set results were also satisfactory. All the validation results indicated that these two models exhibited good stability and predictability.

#### The Major Structure Descriptors of the Best QSAR Model

According to the *t* test value, the most statistically significant descriptor was the polarity parameter/square distance, D_1_. This was an electrostatic descriptor defined as the maximum positive atomic partial charge minus the minimum negative charge divided by their square distance [21]. According to the values listed in Table 1, polarity parameters are only influenced by the number of –COO^−^ and the benzene ring substituent group. The increase of –COO^−^ and the benzene-ring substituent group significantly decreased the value of P″, which favored antibacterial activity against *E. coli*. For example, compound **4** had a P″ value of 0.1249. The charge distribution also changed when a Cl atom was introduced into the benzene ring, which led to a decrease in the P″ value of compound **6** (1.6089 × 10^−3^) [22]. This conclusion is applied in the design of new compounds.

The second descriptor was the FPSA-3 Fractional Charge Weighted Partial Positive Surface Area (PPSA-3/TMSA), D_2_. This descriptor was defined as the fractional charge weighted partial positive surface area, which indicated that an increase in the chain length of cinnamaldehyde compounds would lead to a decrease in the value of the FPSA-3 [23]. According to the *t* test, the decrease of the FPSA promotes antibacterial activity against *E. coli*. Therefore, the chain length should be considered in future designs to increase antibacterial activity.

The third important descriptor was D_3_, an electron density-based descriptor that was the average value of the total bond order of a C atom [24]. The bond order of a C atom reflects the stability of a C–C bond and it also reflects the electron structure. The bond order can determine if electron delocalization occurs between a pair of atoms. A very small bond order decreases electron delocalization and it results in electron flow difficulty. In the optimal QSAR model for *E. coli*, a negative coefficient for D_3_ indicated that a small bond order of the C–C atom resulted in a positive contribution to the antibacterial activity against *E. coli*.

The last descriptor parameter of the best QSAR model against *E. coli* was the relative number of single bonds, D_4_, reflecting the molecular size [25]. In Table 1, the negative correlation coefficient indicates that compounds with small molecular size are more active against *E. coli*.

The optimal QSAR model against *S. aureus* was obtained using the same method. According to the *t* test values, the most important descriptor for the activity of cinnamaldehyde-amino acid compounds against *S. aureus* was the minimum atomic state energy for an H atom (D_5_). This descriptor was a quantum chemical descriptor, and it was related to the state energy of the H atom in a molecule. Low energy allows for a more stable molecule with more H atoms [26]. Equation (3) shows that D_5_ negatively contributed to antibacterial activity against *S. aureus*, wherein an increase in D_5_ would result in a decrease in antibacterial activity.

The second descriptor was the WNSA-1 weighted PNSA (D_6_). WNSA-1 is a quantum-chemical descriptor, which characterizes molecules by molecular shape and electron distribution and is defined in Equation (4):

(4) $$\mathrm{WNSA}-1\;=\frac{\mathrm{PNSA}1\times \mathrm{TMSA}}{1000}$$

where PNSA1 is the partial negatively charged molecular surface area and the TMSA is the total molecular surface area [27]. This descriptor was defined based on the total molecular surface area and charge distribution in the molecule. It indicated the influence of charge distribution on antibacterial activity [27]. According to the optimal model, an increase in WNSA-1 led to a decrease in the antibacterial activity of cinnamaldehyde compounds.

The third descriptor was ESP-RNCS, i.e., the relative amount of negatively charged SA (D_7_). This was a quantum-chemical descriptor of the fraction of the surface area of a molecule that was negatively charged. As shown in Table 1, the descriptors of ESP-RNCS relative to the negatively charged SA were selected from about 400 descriptors, suggesting that the surface area of the molecule had an important influence on the antibacterial activity of CAAS compounds [28].

The last descriptor parameter was the number of Cl atoms (D_8_), a constitutional descriptor. This descriptor is related to molecular polarity. The presence of a Cl atom increases the polarity of the compounds. A previous study showed that most compounds with a Cl atom on the benzene ring exhibited better antibacterial activity than compounds that lacked a Cl atom. Previous studies have also reported that compounds with stronger-electron-withdrawing substituents on the benzene ring showed greater antibacterial activity. This conclusion was also consistent with a research paper on pyrazole derivatives [29]. A Cl-atom substituent on the benzene ring improved the bioactivity.

### 2.2. The Synthesized of Screened Compounds

**Figure 4:**
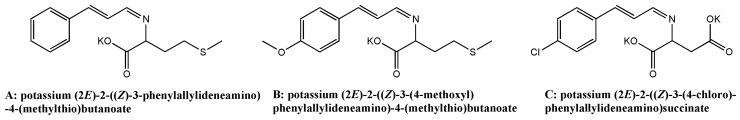
The structures of the screened compounds.

According the structure molecule descriptors of the best QSAR model, three of the 10 cinnamaldehyde-amino acid Schiff bases with good predicted antibacterial activity were selected, synthesized, and the antibacterial activity was then tested. The structures of the three selected compounds are listed in Figure 4, where compound A is used as a bioavailable dietary supplement for ruminant animals to provide essential amino acids and where it also has antimicrobial activity [30]. The structures of the 10 compounds were drawn and the calculated lgAR was listed in the Supplementary Materials, as shown in Figure S1 and Table S1. Three selected compounds, A, B, and C were synthesized, and the antibacterial activity was tested. The structures are listed in Figure 4. The structures of the synthesized compounds were confirmed using FTIR, ^1^H-NMR, ^13^C-NMR, MS, yield and melting point, where the results showed that these were total compounds. Structure characterization results were as follows.

Compound A: potassium (2*E*)-2-((*Z*)-3-phenylallylideneamino)-4-(methylthio)butanoate; Yield: 43.34%; m.p. 287.2 °C; FTIR(cm^−1^): 1578, ν(C=O, C=N, C=C), 753,ν(C_Ar_=H), 694,ν(C_Ar_=H); ^1^H-NMR: δ_H_ (400 MHz, MeOD) 7.96 (1 H, -CH=N, t, *J* 7.0), 7.46 (2 H, Ph-H, dd, *J* 11.2, 4.2), 7.27 (2 H, Ph-H, dd, *J* 5.9, 1.5), 7.06–6.99 (1 H, CH=C, m), 6.86 (1 H, C=CH, dd, *J* 16.0, 8.9), 3.78 (1 H, CH-N-, dd, *J* 8.8, 4.7), 2.50–2.37 (2 H, -CH_2_, m), 2.17–2.10 (1 H, -CH_2_, m), 2.03–1.99 (1 H, -CH_2_, m), 1.98 (3 H, -CH_3_, s); ^13^C NMR (101 MHz, MeOD) δ179.33(-COOK), 165.86(-CH=N), 144.49(-C=C), 130.55(Ph-C), 129.99(Ph-C), 129.44(Ph-C), 128.54(Ph-C), 128.24(C=C-), 76.25(-CH-N), 34.72(-CH_2_), 31.93(-CH_2_), 15.33(-CH_3_). MS (ESI) *m*/*z* calcd. for C_14_H_16_KNO_2_S 301.0, found [M + H]^+^ 301.6.

Compound B: potassium (2*E*)-2-((*Z*)-3-(4-methoxyl)phenylallylideneamino)-4-(methylthio) butanoate; Yield: 55.53%; m.p. 277.3 °C; FTIR(cm^−1^): 1630, ν(C=O,) 1579, ν(C=N, C=C), 1509, ν(C_Ar_=C_Ar_), 824, ν(C_Ar_=H); ^1^H-NMR: δ_H_ (400 MHz, MeOD) 7.96–7.89 (1 H, -CH=N, m), 7.44–7.36 (2 H, Ph-H, m), 7.02–6.92 (1 H, Ph-H, m), 6.86–6.81 (2 H, CH=C, m), 6.73 (1 H, C=CH, dd, *J* 15.9, 9.0), 3.76 (1 H, CH-N-, dd, *J* 8.8, 4.7), 3.71 (3 H, -OCH_3_, d, *J* 5.6), 2.45–2.37 (1 H, -CH_2_, m), 2.31 (1 H, -CH_2_, ddd, *J* 12.9, 8.8, 7.1), 2.13 (1 H, -CH_2_, dddd, *J* 13.7, 9.0, 7.1, 4.7), 1.97 (3 H, -CH_3_, d, *J* 3.7), 1.97–1.91 (1 H, -CH_2_, m); ^13^C NMR (101 MHz, MeOD) δ179.43(-COOK), 166.13(-CH=N), 162.32(Ph-C-O), 144.36(-C=C), 130.12(Ph-C), 125.97(C=C-), 115.43(Ph-C), 75.96(N-CH), 55.98(-OCH_3_), 34.64(-CH_2_), 31.97(-CH_2_), 15.29(-CH_3_); MS (ESI) *m*/*z* calcd. for C_15_H_18_KNO_3_S 331.0, found [M + K]^+^ 370.8.

Compound C: potassium (2*E*)-2-((*Z*)-3-(4-chloro)-phenylallylideneamino)succinate; Yield: 71.90%; m.p. 227.2 °C; FTIR(cm^−1^): 1633, ν(C=O), 1590, ν(C=N, C=C), 1520, ν(C_Ar_=C_Ar_), 815, ν(C_Ar_-H); ^1^H-NMR: δ_H_ (399 MHz, MeOD) 8.08–8.02 (1 H, -CH=N, m), 7.57–7.52 (2 H, Ph-H, m), 7.39–7.35 (2 H, Ph-H, m), 7.08 (1 H, CH=C, d, *J* 16.0), 6.95 (1 H, C=CH, dd, *J* 16.0, 8.7), 3.73 (1 H, CH-N-, dd, *J* 8.3, 4.9), 2.17–2.11 (2 H, -CH_2_, m) [16]; ^13^C NMR (101 MHz, MeOD) δ 179.87(-COOK), 165.43(CH=N), 142.28(-C=C), 135.90(Ph-C-), 130.02(Ph-C), 129.83(Ph-C), 129.44(C=C-), 75.02(CH-N), 15.47(-CH_2_); MS (ESI) *m*/*z* calcd. for C_13_H_10_ClK_2_NO_4_ 357.0, found [M + K]^+^ 395.9.

### 2.3. The Antibacterial Activity of the Screened Compounds

**Figure 5:**
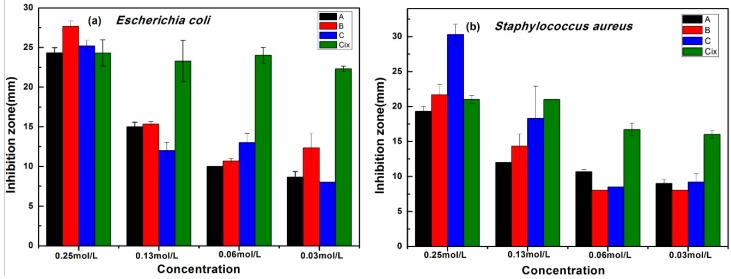
The inhibition zone of the screened compounds against *E. coli* (**a**) and *S. aureus* (**b**).

The diameter of the inhibition zone of new compounds was used to reflect the antibacterial activity, and ciprofloxacin (Cix) was used as the standard. The average and the standard deviation of the inhibition zones of the three compounds are shown in Figure 5. As shown in Figure 5a, the three screened compounds exhibited excellent antibacterial activity. The diameter of the inhibition zones of compounds A, B, and C was 24.33, 27.67, and 24.67 mm, respectively, at 0.25 mol/L, which was slightly higher than that of the drug ciprofloxacin (Cix: 24.33 mm) against *E. coli*. An obvious decrease in the diameter of the inhibition zone for the screened compounds was observed as the test concentration was decreased. Even at the minimal tested concentration of 0.03 mol/L, the screened compounds still exhibited good antibacterial activity. Similar inhibition of the screened compounds was observed for *S. aureus*, as shown in Figure 5b. A comparison of Figure 5a,b, reveals that *S. aureus* was more resistant to the screened compounds than *E. coli*, and the screened compounds possessed comparable antibacterial activity to ciprofloxacin against *E. coli* and *S. aureus*.

The antibacterial activity rates of the compounds were calculated using Equation (5) and are listed in Table 4. The Exp.lgAR values were close to the Cal.lgAR of the screened compounds, and all the Cal.lgAR values were less than the Exp.lgAR, except for compound C. Overall, the two QSAR models of cinnamaldehyde-schiff base compounds showed good predictability.

## 3. Materials and Methods

### 3.1. Materials

Trans-cinnamaldehyde was produced by the Zhenxing Spices Oil Refinery of Ji’an City, China. All the solvents and amino acids were analysis level trans-*p*-chloro-cinnamaldehyde and trans-*p*-methoxy-cinnamaldehyde. The test bacteria were the gram-positive bacteria *Staphylococcus aureus* (*S. aureus*) and the gram-negative bacteria *Escherichia coli* (*E. coli*), where samples were provided by the Chinese Center of Industrial Culture Collection (CICC), Beijing, China. All the microorganisms were cultured on beef extract tryptone agar at 37 °C for 12 h.

### 3.2. Method

#### 3.2.1. Determination of Antibacterial Activity

The paper disc method described in Reference [30] was used to measure the antibacterial activity of the new synthesized cinnamaldehyde compounds. Petri dishes and tweezers were packed with wasted newspaper and sterilized at 160 °C for 2 h in an oven. Sterilized 0.9 wt% NaCl solution and 2% beef extract tryptone agar (BTA) medium were prepared. All the materials used for the microorganism tests were UV sterilized for 20 min before use. The BTA medium was poured into petri dishes and allowed to solidify. Then, 125 μL of bacterial suspension that was diluted 10 × 10^4^ times was spread onto the surface of the agar. Next, a paper disc was saturated with the test compounds (0.125 mol/L) and placed in the center of the agar plate. The plate was then transferred to a constant temperature incubator and incubated for 12 h at 37 °C. Next, the diameter of inhibition zones was measured and used to express the antibacterial activity. In this test, cinnamaldehyde served as the control. Cix, as described in Reference [31], was used as the standard drug to evaluate the antibacterial activity of the screened compounds. The AR was expressed as the following equation:

(5) $$\mathrm{AR}\left(\%\right)=\left(\frac{d}{d_{c}}\right)\times 100\%$$

In the above equation, *d* and *d_c_* are the diameter of the inhibition zone of the test compounds and the control compound, respectively. All the samples were analyzed in triplicate, and the inhibition zones were presented as mean values.

#### 3.2.2. Establishing QSAR Models

3D molecular structures of 21 cinnamaldehyde compounds were prepared and initial optimization was performed using the Chembio 3D software. The most stable configurations of all the compounds were optimized at the AMI/destricted HF level using the AMPAC Agui 9.2.1 software. In this paper, the antibacterial activity rates of cinnamaldehyde compounds were used as the properties. After an additivity calculation for the AR, the logarithmic value of AR (lgAR) was used as the variable to establish the QSAR models. The structure-data files were inputted into Codessa 2.7.16 software to calculate the molecule descriptor. Next, the “best multi-linear regression” was determined to calculate the relationship between bioactivity and the descriptors [32]. The structure of the compounds used to establish the model is listed in Figure 1.

#### 3.2.3. Validation of the QSAR Models

The entire dataset was split into training and test sets. The training set model was established as a “multi-linear regression” using the same descriptor of the best QSAR model. The training set model was used to predict the antibacterial activity of the test set compounds. The statistical results of the training set and the test set, including the correlation coefficient (R^2^), Fisher value (F), and standard deviation (S^2^), were used to evaluate the predictability and stability of the best QSAR model and then cross-validation was conducted, as in Reference [33].

The first and second grouping methods employed corresponding to cross-validation and “leave one out” validation. First, 21 compounds were randomly divided into 3 groups, designated a(**1**, **5**, **9**, **10**, **14**, **18**, **19**), b(**2**, **4**, **8**, **12**, **13**, **17**, **21**), and c(**3**, **6**, **7**, **11**, **15**, **16**, **20**). Every group combined as a training set, designated as A, B, and C, with the remainder designated as the test set. This grouping method was used for the cross-validation. Second, according to the number of compounds, one fourth of the compounds were placed in a test set labeled as group d(**4**, **8**, **12**, **16**, **20**), and the other compounds were labeled as group D, and set as the training set. All the validation results are listed in Table 3.

#### 3.2.4. Synthesis Methods of Screened Compounds

Equal molar mass of amino acids and KOH were added into ethanol and stirred at 50 °C until the amino acids dissolved. Then, 1.2 equivalent molar mass of cinnamaldehyde was added drop-by-drop over 30 min at room temperature with constant stirring. After addition, the mixtures were constantly stirred and allowed to react for two hours. After reaction, the solvent was evaporated at 35 °C until precipitate formed. The precipitate was then washed three times to remove the extra cinnamaldehyde, as described in Reference [16]. The washed precipitate was the prepared compound. FTIR, ^1^H-NMR, ^13^C-NMR, MS, and melting point were then used to determine the structures of the compounds.

## 4. Conclusions

Two best QSAR models of 21 cinnamaldehyde compounds were built and validated. The two QSAR models where the models with satisfactory statistical parameters. The QSAR models provided some insight into the structural character and its relationship with antibacterial activity against *E. coli* and *S. aureus*. Based on the QSAR models, three cinnamaldehyde Schiff base compounds were screened, synthesized, and the structures characterized. The antibacterial activity test of the new synthesized compounds revealed that the QSAR models had good predictability. 

## Figures and Tables

**Table 1:** The antibacterial activity rates (AR) and the value of molecular descriptors for the best QSAR model against *E. coli* and *S. aureus*.

ID	*Escherichia coli*						*Staphylococcus aureus*					
	AR	lgAR	P″, D_1_	FPSA3, D_2_	ABOC, D_3_	RNSB, D_4_	AR	lgAR	MASEH, D_5_	WNSA1, D_6_	RNCS, D_7_	NACl, D_8_
**1**	163.64	2.2139	5.0339 × 10^−3^	0.0098	1.0927	0.6875	91.11	1.9596	−7.2770	127.7249	13.5586	0.0000
**2**	106.36	2.0268	2.7696 × 10^−3^	0.0142	1.0782	0.7222	88.89	1.9488	−7.3330	126.9269	4.5587	0.0000
**3**	157.27	2.1967	2.1257 × 10^−3^	0.0111	1.0915	0.6875	222.22	2.3468	−7.3530	172.4734	11.4494	1.0000
**4**	72.73	1.8617	0.1249	0.0111	1.1075	0.6250	88.89	1.9488	−7.3180	87.3458	10.0842	0.0000
**5**	72.73	1.8617	2.3127 × 10^−3^	0.0147	1.1561	0.6786	88.89	1.9488	−7.3170	90.6090	6.6390	0.0000
**6**	163.64	2.2139	1.6089 × 10^−3^	0.0113	1.1059	0.6250	122.22	2.0872	−7.3100	112.3508	16.1548	1.0000
**7**	72.73	1.8617	0.1249	0.0120	1.0933	0.6667	96.67	1.9853	−7.3840	89.1705	8.1225	0.0000
**8**	72.73	1.8617	2.5943 × 10^−3^	0.0148	1.1732	0.7097	103.33	2.0142	−7.3640	119.4079	6.6248	0.0000
**9**	113.64	2.0555	1.7586 × 10^−3^	0.0112	1.1650	0.6667	355.56	2.5509	−7.3800	138.2133	16.1512	1.0000
**10**	75.45	1.8777	0.1249	0.0106	1.0584	0.7692	88.89	1.9488	−7.3180	89.6997	12.0427	0.0000
**11**	127.27	2.1047	2.8601 × 10^−3^	0.0127	1.0487	0.7907	88.89	1.9488	−7.3340	110.4771	5.3309	0.0000
**12**	161.82	2.2090	1.9326 × 10^−3^	0.0097	1.0579	0.7692	141.11	2.1496	−7.3170	132.7288	12.8490	1.0000
**13**	72.73	1.8617	0.1250	0.0117	1.0725	0.7273	88.89	1.9488	−7.3350	108.6156	5.7232	0.0000
**14**	88.18	1.9454	3.0117 × 10^−3^	0.0140	1.1118	0.7568	88.89	1.9488	−7.3360	115.2242	5.8626	0.0000
**15**	139.09	2.1433	2.0303 × 10^−3^	0.0106	1.0716	0.7273	174.44	2.2417	−7.3300	139.9473	14.6326	1.0000
**16**	177.27	2.2486	4.2908 × 10^−3^	0.0107	1.1169	0.6053	127.78	2.1065	−7.3210	134.7791	13.4207	0.0000
**17**	111.82	2.0485	3.8575 × 10^−3^	0.0136	1.1250	0.6429	103.33	2.0142	−7.3480	157.7156	3.6624	0.0000
**18**	118.18	2.0726	4.2916 × 10^−3^	0.0110	1.1164	0.6053	144.44	2.1597	−7.3080	172.0321	7.1417	1.0000
**19**	81.82	1.9128	0.1251	0.0112	1.0518	0.6053	88.89	1.9488	−7.3450	111.6619	6.6481	0.0000
**20**	125.45	2.0985	2.6664 × 10^−3^	0.0135	1.1063	0.6429	92.22	1.9648	−7.2920	175.5388	4.9846	0.0000
**21**	227.27	2.3565	1.9517 × 10^−3^	0.0106	1.0512	0.6053	277.78	2.4437	−7.3320	154.3464	13.3231	1.0000

Abbreviation of descriptors: P″—Polarity parameter/square distance; FPSA3—Fractional charge partial positive surface area; ABOC—Average bond order of a C atom; RNSB—Relative number of single bonds; MASEH—Min atomic state energy for an H atom; WNSA1—weighted partial negatively charged molecular surface area; RNCS—Relative negative charged surface area; NACl—Number of Cl atoms.

**Table 2:** The optimal QSAR model for cinnamaldehyde-amino acid Schiff base compounds against *E. coli* and *S. aureus*.

No	X	ΔX	*t* test Value	Name of Descriptor
*E. coli* model: R^2^ = 0.9354, F = 57.96, and s^2^ = 0.0020				
0	5.0709	4.0665 × 10^−1^	14.8035	Intercept
1	−2.5685	1.9802 × 10^−1^	−13.5819	Polarity parameter/square distance, D_1_
2	−4.1057 × 10	7.4151	−5.5369	FPSA3 Fractional PPSA (PPSA-3/TMSA) [Zefirov’s PC], D_2_
3	−1.7850	3.4648 × 10^−1^	−5.1519	Avg bond order of a C atom, D_3_
4	−7.2082 × 10^−1^	1.8119 × 10^−1^	−3.9783	Relative number of single bonds, D_4_
*S. aureus* model: R^2^ = 0.8946, F = 33.94, and s^2^ = 0.0043				
0	−1.8664 × 10	4.3463	−4.2942	Intercept
1	−2.7525	5.8814 × 10^−1^	−4.6799	Min atomic state energy for an H atom, D_5_
2	2.6523 × 10^−3^	6.9487 × 10^−4^	3.8170	WNSA-1 Weighted PNSA(PNSA1 × TMSA/100)[Quantum-Chemical PC], D_6_
3	1.9642 × 10^−2^	5.2856 × 10^−3^	3.7162	RNCS Relative negative charged SA(SAMNEG × RNCG)[Zefirov’s PC], D_7_
4	1.1873 × 10^−1^	5.2091 × 10^−2^	2.2794	Number of Cl atoms, D_8_

**Table 3:** The internal validation results of best QSAR models.

Training Set	N	R^2^ (fit)	F (fit)	s^2^ (fit)	Test Set	N	R^2^ (pred)	F (pred)	s^2^ (pred)
Validation for the model of *E. coli*									
A	14	0.9456	39.08	0.0020	c	7	0.8759	42.36	0.0171
B	14	0.8951	19.20	0.0030	b	7	0.9826	338.07	0.0096
C	14	0.9816	120.20	0.0007	a	7	0.7800	21.27	0.0227
Average	14	0.9408	59.49	0.0019	Average	7	0.8795	133.9	0.0165
D	16	0.9262	34.52	0.0023	d	5	0.9620	101.25	0.0162
Validation for the model of *S. aureus*									
A	14	0.9339	31.79	0.0037	c	7	0.6790	12.69	0.0322
B	14	0.9121	23.35	0.0044	b	7	0.8410	31.75	0.0273
C	14	0.8754	15.81	0.0046	a	7	0.9089	59.84	0.0259
Average	14	0.9071	23.65	0.0042	Average	7	0.8096	34.76	0.0282
D	16	0.8975	24.08	0.0056	d	5	0.8029	16.29	0.0175

**Table 4:** The antibacterial activity rates and comparison of predicted values and experiment values for screened compounds A, B, and C at a concentration of 0.125 mol/L.

No.	*Staphylococcus aureus*				*Escherichia coli*			
	Exp.AR	Exp.lgAR	Cal.lgAR	Error	Exp.AR	Exp.lgAR	Cal.lgAR	Error
A	133.33	2.1249	1.8365	0.2884	136.36	2.1347	1.8831	0.2516
B	159.22	2.2020	2.0876	0.1144	136.36	2.1347	1.9323	0.2024
C	203.33	2.3082	2.3131	−0.0049	109.09	2.0378	2.1099	−0.0721

## Supplementary Materials

The 3D-structures of screened compounds are available in MOL formats file.
