# Supplementary material for: Screening, Synthesis, and QSAR Research on Cinnamaldehyde-Amino Acid Schiff Base Compounds as Antibacterial Agents
Source: Molecules. 2018 Nov 20;23(11):3027. doi: 10.3390/molecules23113027 (PMC6278255; doi:10.3390/molecules23113027)
Supplement: Supplementary file 1 [file molecules-23-03027-s001.zip › Supple/Supplementary materials 4-The experimental lgAR and Calculated lgAR of screened compounds.pdf]

Supplementary Information

Article

Screening, Synthesis, and QSAR Research on Cinnamaldehyde-Amino Acid Schiff Base Compounds as Antibacterial Agents

Hui Wang [1,2], Mingyue Jiang [2], Fangli Sun [1,*], Shujun Li [2,*], Chung-Yun Hse [3] and Chunde Jin [1]

[1] School of Engineering, Zhejiang A&F University, Lin'an 311300, China; wh-snowis@outlook.com (H.W.); jincd@zafu.edu.cn (C.J.)

[2] Key Laboratory of Bio-Based Material Science and Technology of the Ministry of Education, Northeast Forestry University, Harbin 150040, China; jiangmingyue@nefu.edu.cn

[3] Southern Research Station, USDA Forest Service, Pineville, LA 71360, USA; chse@fs.fed.us

* Correspondence: sun-fangli@163.com (F.S.); lishujun@nefu.edu.cn (S.L.); Tel.:+86-137-3813-1516 (F.S.); +86-451-8219-1742 (S.L.)

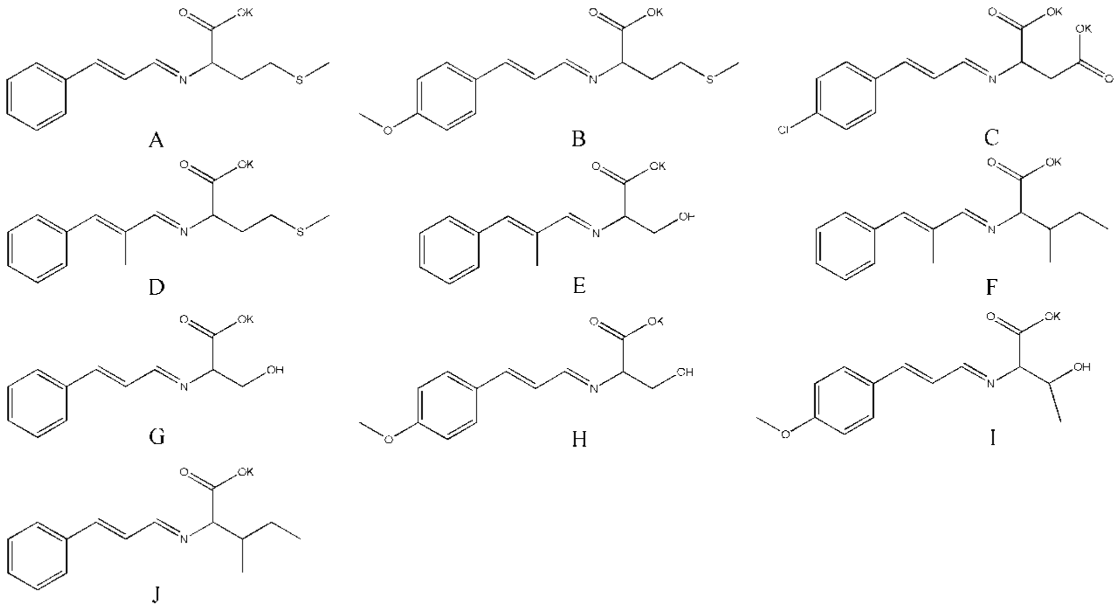

Figure S1. The designed compounds using best QSAR models

Table S1. The calculated lgAR of screened compounds according to the best QSAR models

No.	Cal. lgAR against *E.coli*	Cal.lgAR against *S.aureus*
A	1.8831	1.8365
B	1.9323	2.0876
C	2.1099	2.3131
D	1.8545	1.9806
E	1.8501	0.8467
F	1.8626	1.9770
G	1.9012	0.8149
H	1.8226	1.1254
I	2.0131	1.9275
J	1.8697	1.8426
